# Indirect mitral annuloplasty using Carillon device after conduction system pacing resynchronization in patient with severe mitral regurgitation: a case report

**DOI:** 10.1186/s12872-026-05682-3

**Published:** 2026-05-13

**Authors:** Abdelrahman Elhakim, Mohamed Elhakim, Osama Bisht, Peter W. Radke, Mohammed Saad

**Affiliations:** 1 Schleswig-Holstein University Hospital-Kiel, Arnold-Heller-Street 3, Kiel, 24105 Germany; 2 Intensive Care Medicine department, The Royal Prince Alfred Hospital, 50 Missenden Rd, Camperdown, , Sydney, NSW 2050 Australia; 3 Interventional Cardiology Consultant , Coswig Herat Center, Coswig, Germany; 4 Cardiology Department, Schoen Hospital Neustadt in Holstein, Am Kiebitzberg 10, Neustadt in Holstein, 23730 Deutschland

**Keywords:** Secondary mitral regurgitation, Indirect mitral annuloplasty, Cardiac resynchronization therapy, Conduction system pacing, Case report

## Abstract

**Background:**

The prevalence of secondary mitral regurgitation (SMR) is increasing due to the aging population. SMR management is a complex disease because at least 2 pathologies are involved. SMR and severely reduced EF are independent risk factors for adverse outcomes.

**Case summary:**

We present the case of a 57-year-old man who presented with dyspnea NYHA III. On echocardiography, a combination of SMR and DCM with EF of 20% were diagnosed. Indirect mitral annuloplasty after CRT-CSP was performed based on the patient’s clinical profile and high surgical repair risk. The 3-month follow-up was uneventful.

**Discussion:**

Mitral valve repair techniques in ventricular SMR with severely reduced EF are controversial due to ongoing debates about long-term repair durability in the setting of left ventricular remodeling. Percutaneous strategies are an alternative for high-risk surgical patients and their indication expanded for more complex anatomy and patient´s comorbidity.

**Conclusion:**

Indirect mitral annuloplasty with the Carillion device in patients with CRT-CSP due to low EF and severe mitral regurgitation is feasible for the treatment of these combined pathologies and allows more physiological pacing and future M-TEER.

**Graphical abstract:**

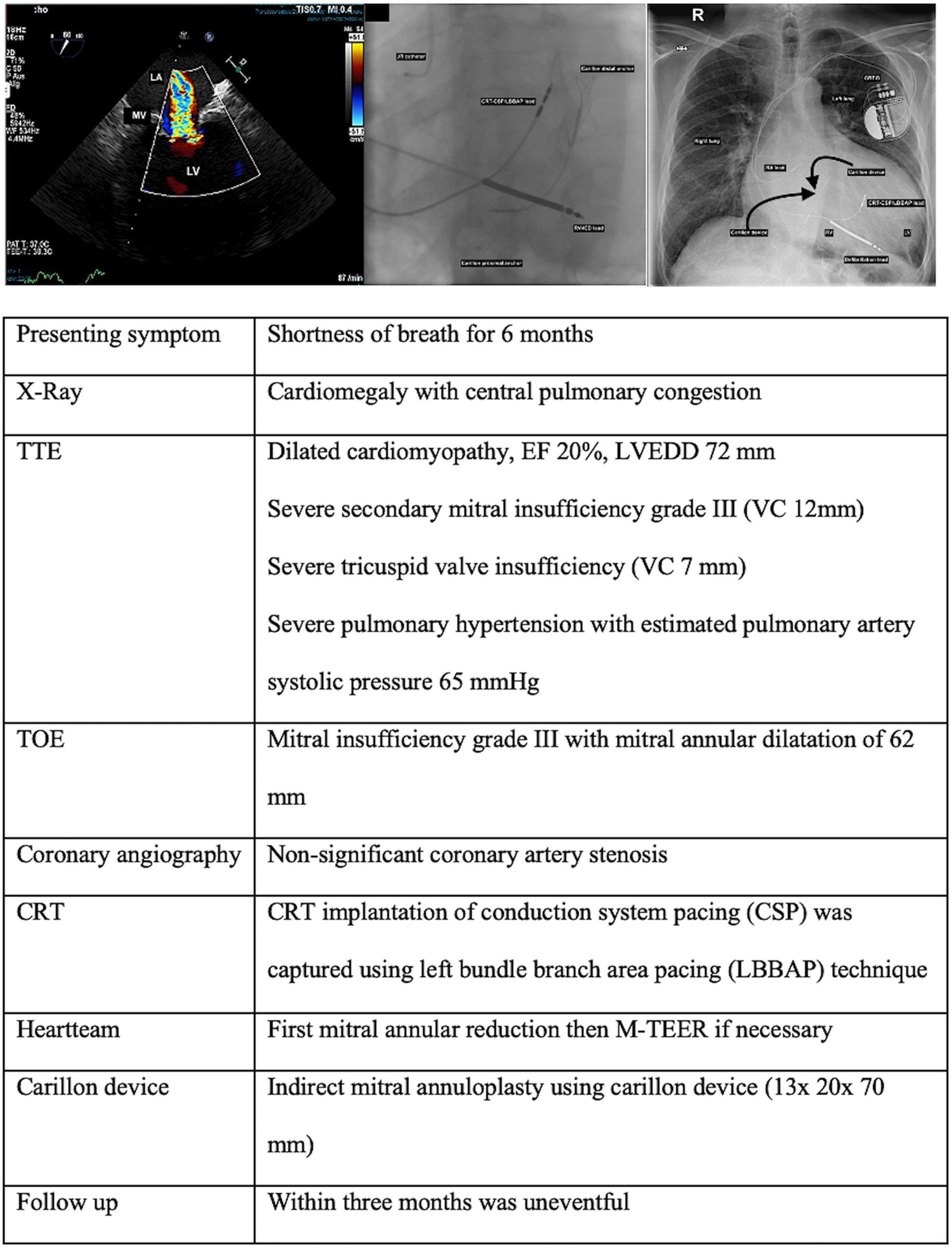

## Introduction

The prevalence of mitral regurgitation (MR) is increasing due to the aging population and comorbidities. Primary MR is a disease with anatomic abnormalities of the mitral valve that cause MR. Timely mitral valve surgery remains the gold standard treatment in low-risk surgical patients.

Secondary mitral regurgitation (SMR) is a considerably more complex disease because at least 2 pathologies are involved. It occurs as a result of left atrioventricular dilatation and maleficent remodeling. It is a common and undertreated form of MR and is associated with a poor prognosis [1].

Patients with SMR often present with symptoms of dyspnea, leading to worsening functional capacity, hospitalization, and even death. Guideline-directed medical therapy (GDMT) for heart failure and treatment of reversible causes can reduce MR in many cases and improve outcomes after confirmation of SMR [1].

In addition, the implantation of cardiac resynchronization therapy (CRT), if indicated, can help reduce SMR by reverse left ventricular (LV) remodeling, but some patients continue to have SMR [2].

A definitive surgical approach in low-to intermediate-risk surgical patients can be considered, whereas percutaneous strategies remain an alternative for high-risk surgical patients [1]. Mitral transcatheter edge-to-edge repair (M-TEER) is safe and effective. The Matterhorn Trial concluded that M-TEER was noninferior in terms of clinical safety and efficacy compared with surgery with a short-term follow-up of 30 days [3]. However, long-term follow-up is ongoing.

Percutaneous indirect mitral ring annuloplasty using the Carillon device is also an option for SMR. The Carillon device utilizes the relationship between the coronary sinus and mitral annulus to perform an “indirect” annuloplasty. The REDUCE-FMR trial, which included patients with LV-EF < 50%, an LV end-diastolic diameter > 55 mm, observed trends toward improvement in the mean 6MWT distance and NYHA class with a low rate of complications [4].

The main limitation of the Carillon device in SMR is the preimplantation of CRT, which can cause lead damage. In such a scenario, CRT patient`s should be excluded or treated first with a Carillon device, then CRT, which is against the guidelines` recommendation. Some operators implant Carillon after CRT as off-label use with an increased risk of lead damage, whereas others try to implant CRT-lead in small or middle cardiac veins to allow Carillon implantation later. In such a scenario, CRT pacing may be less effective than lead implantation in the posterolateral vein.

We report a case of severe SMR with low EF in which successful carillon device implantation after CRT implantation of conduction system pacing (CSP) was captured using the left bundle branch area pacing (LBBAP) technique.

## Case presentation

A 57-year-old man with a history of chronic heart and renal failure initially presented with shortness of breath for 6 months. The patient was known to have paranoid schizophrenia and alcohol abuse. On physical examination, a systolic murmur was noted at the left fifth intercostal space. Electrocardiography revealed sinus rhythm and complete left bundle branch block (LBBB). Laboratory parameters revealed NT pro-BNP levels of 22,239 pg/ml and creatinine of 1.4 mg/dl.

**Fig. 1:**
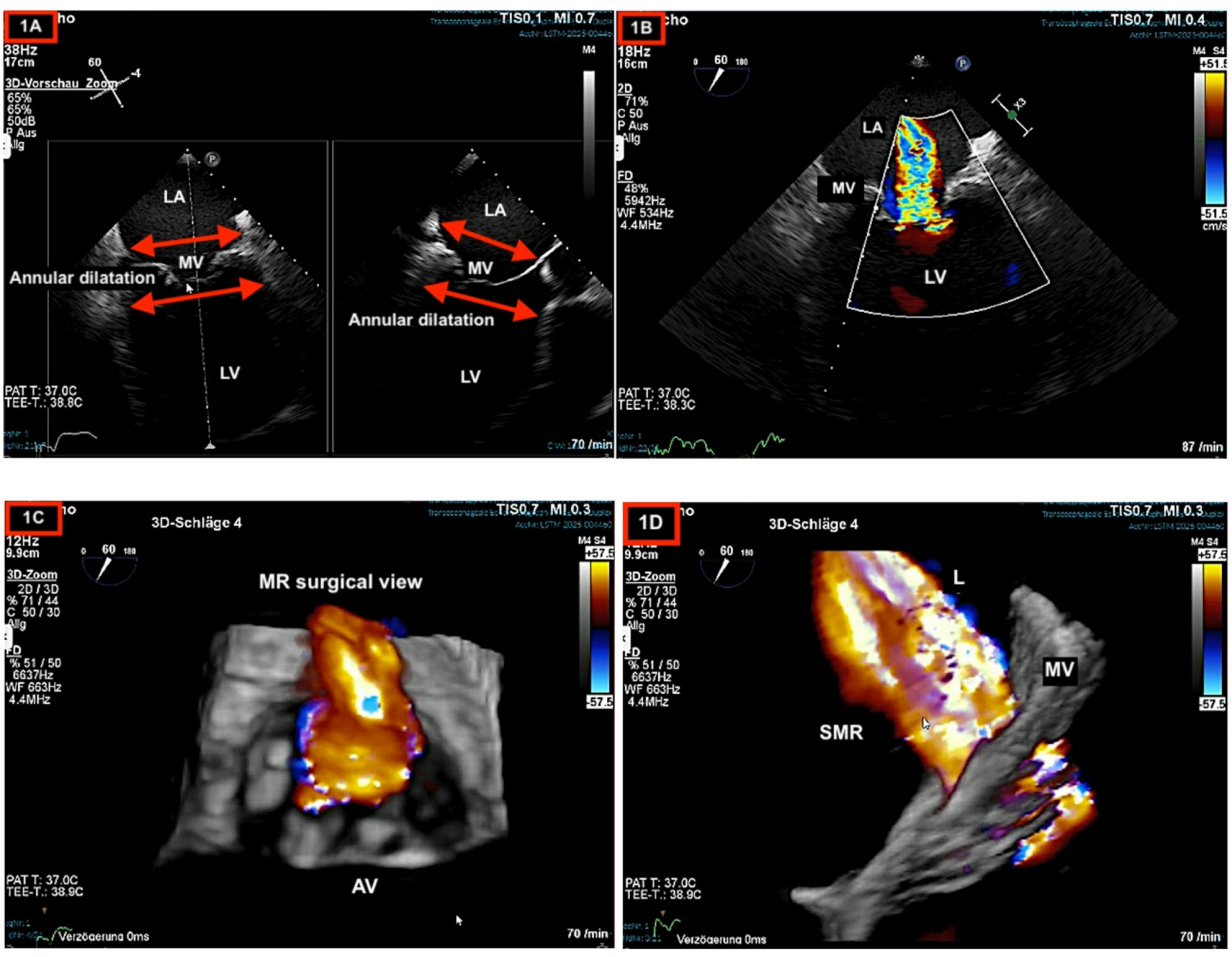
Transesophageal echocardiography demonstrating (**A**) the presence of atrioventricular ring dilatation, (**B**) sever central mitral regurgitation, (**C**) three-dimensional mitral regurgitation in surgical view, (**D**) three-dimensional mitral regurgitation in lateral view. *LA* Left atrium, *MV *Mitral valve, *LV* Left ventricle, *AV* Aortic valve, *SMR* Secondary mitral regurgitation

A transthoracic echocardiogram (TTE) revealed an LV end-diastolic diameter of 72 mm, and an EF of 20%. Severe SMR (Vena contracta (VC)12 mm). Transoesophageal echocardiography (TOE) confirmed the diagnosis of severe SMR due to mitral annular dilatation of 62 mm (Fig. 1A-D). Guideline-directed medical therapy was administrated, including an angiotensin receptor-neprilysin inhibitor (ARNI), a beta-blocker, a mineralocorticoid receptor antagonist (MRA), a sodium-glucose cotransporter 2 (SGLT2) inhibitor, and Aspirin therapy. Three months later, echocardiography revealed no improvement in LV function and mitral valve insufficiency.

The patient underwent diagnostic catheterization, which revealed non-significant coronary artery stenosis. We upgraded the 2-chamber ICD to a CRT-D system as CSP/LBBAP (Biotronik Rivacor 3 HF-T) with placement of the third lead in the proximal septal position, and QRS duration reduction to 90 ms was achieved. The 3-month follow-up showed worsening functional capacity under minimal exertion.

After meticulous evaluation by the heart team, biventricular assist devices and heart transplantation were excluded due to the risk of paranoid schizophrenia and alcohol abuse. The patient refused surgical mitral repair. There was uncertainty regarding the M-TEER because of the 62 mm mitral ring dilatation. Therefore, we decided to perform indirect mitral ring annuloplasty using a Carillon device (Cardiac Dimensions, WA, USA). Upgrading with M-TEER is possible in patients without improvement of symptoms. In addition, the previously implanted CRT-CSP will not interact with the Carillon device.

The procedure was performed with the consensus of the heart team and after patient´s consent.

**Fig. 2:**
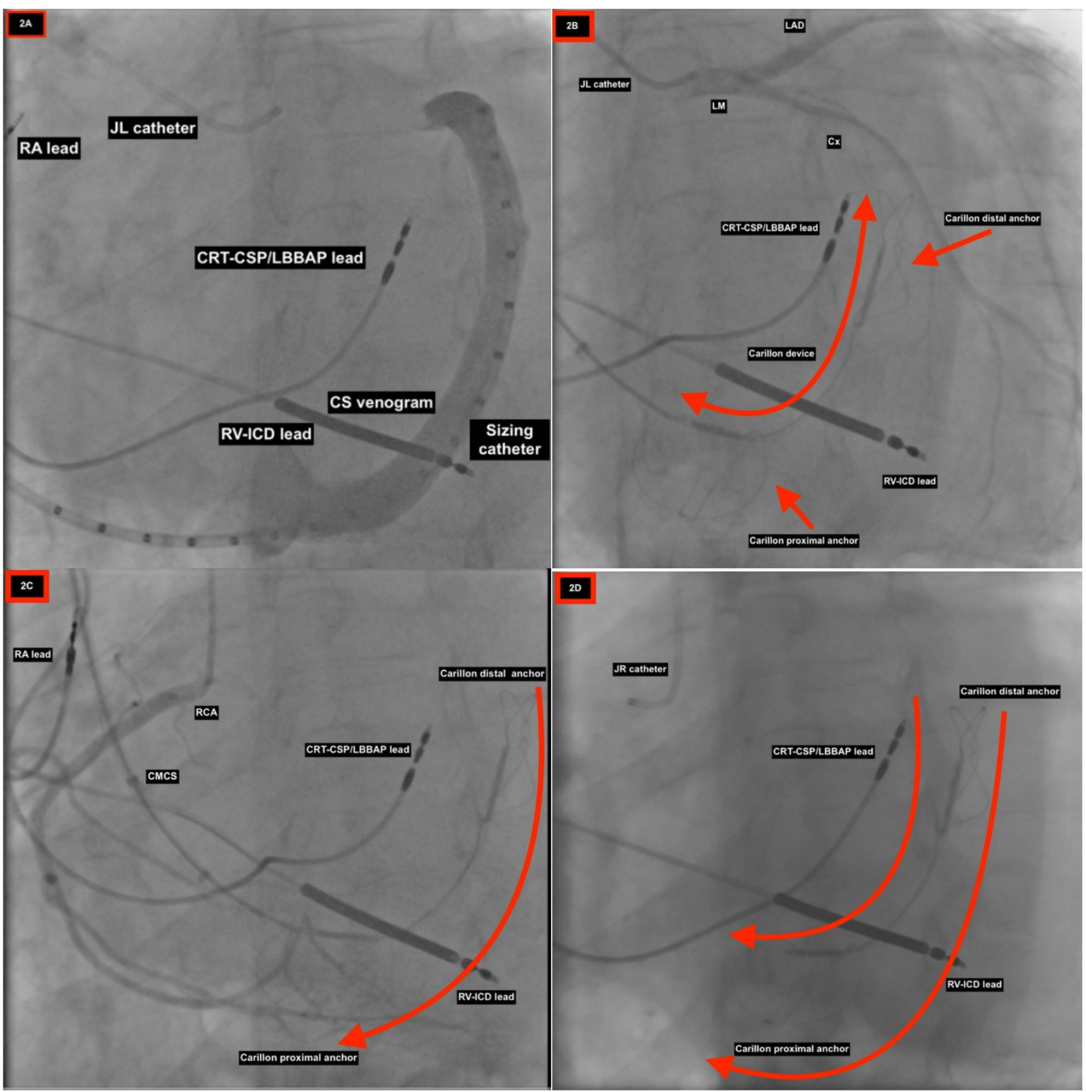
Cine imaging demonstrating (**A**) venogram of coronary sinus with sizing catheter inside, (**B**) Carillon device in relation to circumflex coronary artery, 3) Carillon device in relation to RCA coronary artery, (**D**) Carillon device in relation to CRT-CSP/LBBAP lead. *JL* Judkin left, *RA* Right atrium, *RV* Right ventricle, *RCA* Right coronary artery, *LV* Left ventricle, *ICD* Implantable cardioverter-defibrillator, *CRT* Cardiac resynchronization therapy, *CSP *Conduction system pacing, *LBBAP* Left bundle branch area pacing, *CMCS *Carillon Mitral Contour System

**Fig. 3:**
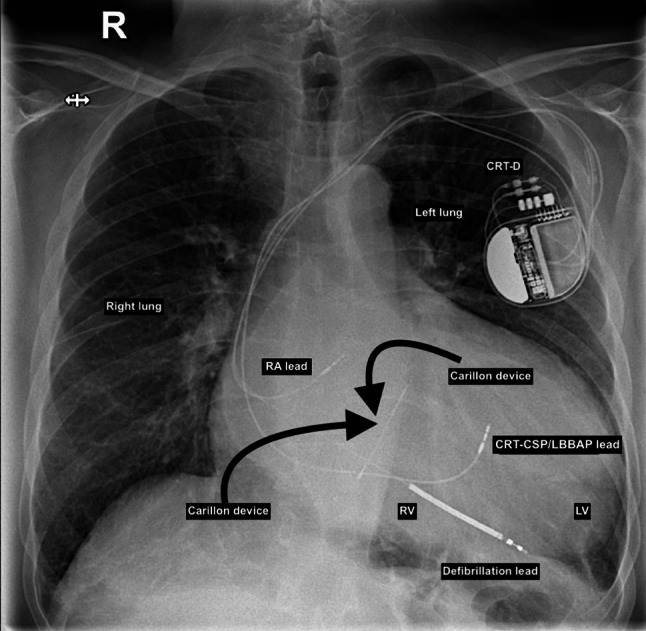
Chest X-ray cardiomegaly with CRT-CSP/LBBAP and Carillon device implantation in coronary sinus. *RA *Right atrium, *RV *Right ventricle, *LV *Left ventricle, *CRT *Cardiac resynchronization therapy, *CSP *Conduction system pacing, *LBBAP *Left bundle branch area pacing

After cannulation of the radial artery for coronary artery injections with a 6-F sheath and the right internal jugular vein with a 10-F sheath, 5000 U heparin was administered. Baseline left and right coronary arteriograms were performed to record the CS ostium location during the venous phase of the injection. Then, a 0.035 soft-tip guidewire was inserted to cannulate the coronary sinus (CS). We advanced the guidewire, diagnostic catheter, and Carillon Mitral Contour System (CMCS) delivery catheter through the CS into the anterior interventricular vein (AIV). After that, we advanced the sizing catheter and performed a contrast venogram during the LAO/caudal 20/30 angulation. At the same time, we performed arteriograms to determine the relationship between the coronary arteries and the CS. After removing the sizing catheter, we determined the implant target zones by considering the available vein length, vein geometry, and coronary artery location. The implant device (13 × 20 × 70 mm) was selected. Deployment of the distal anchor at the target location and retraction of the delivery catheter to the proximal anchor crimp tube`s distal end. At this stage, as guided by fluoroscopy, we placed gradual tension on the implant by gently pulling on the CMCS delivery catheter until the proximal anchor crimp tube was at the proximal anchor target location in the CS. The proximal anchor was deployed at the target position in the CS. Coronary arteriogram showed no significant impact on the left and right coronary arteries. Finally, the proximal Anchor was released, and the CMCS delivery catheter was withdrawn (Fig. 2A-D) and (Fig. 3).

At the end of the procedure, the VC was reduced from 12 to 8 mm in the TTE. After sheath removal, hemostasis of internal jugular vein was achieved via manual compression without complications.

The patient was discharged after 2 days. The 6-month follow-up revealed an improvement in the quality of life and dyspnea symptoms to NYHA I-II with LV-EF of 30%, and no re-hospitalization events. occurred. However, the MR-VC remained 8 mm. We recommend further medical therapy with consideration of M-TEER only by symptom deterioration.

## Discussion

We report a case of successful indirect mitral annuloplasty using the Carillion device in a patient with previously implanted CRT-CSP due to HFrEF and severe SMR with improvement of symptoms and without complications.

The current ESC guideline recommends M-TEER for symptomatic patients with ventricular SMR, reduced LV function, and not eligible for surgery as class IIa and for patients with severe atrial SMR as class IIb whereas indirect mitral annuloplasty using the Carillion device has no recommendation [5].

Although percutaneous strategies are an alternative for high-risk surgical patients, the main limitation of M-TEER in this case was severe LV annular dilatation with large coaptation gab. The carillon device facilitates mitral annular ring diameter reduction, thereby paving the way for the future feasibility of M-TEER. The main limitations of this approach are the presence of primary mitral regurgitation and moderate to severe mitral annular calcification [5].

In addition, the CRT-CSP implantation technique was selected in which the third lead was implanted to the proximal septum and not in the CS. It captures LBBAP and is associated with better ventricular synchrony [5]. 

The advantages of CRT-CSP implantation are as follows:

Enables more physiological pacing. Thus, MR reduction is possible.

This approach facilitated Carillon device`s future implantation.

The proposed method provides a universal algorithm for MR repair, regardless of the technique used.

Facilitates the adoption of guidelines to improve outcomes.

The septal hematoma, lead macro-dislodgment, tricuspid regurgitation, less favorable RV synchrony, and lack of long-term data are major limitations of CSP.

Finally, more cases are required to evaluate the safety and feasibility of this approach in this patient group.

## Conclusion

Percutaneous mitral valve repair techniques are an alternative for high-risk surgical patients, and their indications are expanded for more complex anatomy and patient´s comorbidity.

Indirect mitral annuloplasty with the Carillion device is feasible for the treatment of these combined pathologies in patients with previously implanted CRT-CSP due to low EF and severe SMR.

## Data Availability

All data related to the case are available on request.

## Abbreviations

AIV: Anterior interventricular vein
CMCS: Carillon Mitral Contour System
CRT: Cardiac resynchronization therapy
CS: Coronary sinus
CSP: Conduction system pacing
ECG: Electrocardiogram
EF: Ejection fraction
GCV: Great cardiac vein
GDMT: Guideline-directed medical therapy
LBBB: Left bundle branch block
MR: Mitral regurgitation
M-TEER: Mitral-transcatheter edge to edge repair
SMR: Secondary mitral regurgitation
TOE: Transoesophageal echocardiography
TTE: Transthoracic echocardiography
